# Potential Therapeutic Application of Regulatory T Cells in Diabetes Mellitus Type 1

**DOI:** 10.3390/ijms23010390

**Published:** 2021-12-30

**Authors:** Iwona Ben-Skowronek, Joanna Sieniawska, Emilia Pach, Wiktoria Wrobel, Anna Skowronek, Zaklina Tomczyk, Iga Rosolowska

**Affiliations:** Department of Paediatric Endocrinology and Diabetology with Endocrine-Metabolic Laboratory, Medical University in Lublin, Prof. A. Gebala Street 6, 20-093 Lublin, Poland; joanna.sieniawska87@gmail.com (J.S.); emilia.k.pach@gmail.com (E.P.); wiktoriaKW_97@interia.pl (W.W.); ania.skowron97@gmail.com (A.S.); zaklina.tomczyk96@gmail.com (Z.T.); iga.rosolowska@gmail.com (I.R.)

**Keywords:** T regulatory cells, diabetes mellitus type 1

## Abstract

The autoimmune reaction against the beta cells of the pancreatic islets in type 1 diabetes mellitus (T1DM) patients is active in prediabetes and during the development of the clinical manifestation of T1DM, but it decreases within a few years of the clinical manifestation of this disease. A key role in the pathogenesis of T1DM is played by regulatory T cell (Treg) deficiency or dysfunction. Immune interventions, such as potential therapeutic applications or the induction of the Treg-cell population in T1DM, will be important in the development of new types of treatment. The aim of this study was to evaluate innovative immune interventions as treatments for T1DM. After an evaluation of full-length papers from the PubMed database from 2010 to 2021, 20 trials were included for the final analysis. The analysis led to the following conclusions: Treg cells play an important role in the limitation of the development of T1DM, the activation or application of Tregs may be more effective in the early stages of T1DM development, and the therapeutic use of Treg cells in T1DM is promising but requires long-term observation in a large group of patients.

## 1. Introduction

T1DM is becoming widespread, with approximately 490,000 children affected worldwide [1]. T1DM is an autoimmune disease that leads to the destruction of insulin-secreting pancreatic beta cells. Insulin is an important anabolic hormone that affects glucose, lipid, protein, and mineral metabolism, as well as growth. Insulin, via insulin receptors, allows glucose to enter muscle and adipose cells; it stimulates the liver to store glucose as glycogen and synthesize fatty acids, and it stimulates the uptake of amino acids, inhibits the breakdown of fat in adipose tissue, and stimulates the uptake of potassium into cells. Patients with T1DM require life-long insulin replacement therapy. The selective destruction of insulin-producing pancreatic beta cells underlies T1DM, which is a genetically determined multifactorial disease. Knowledge on the pathogenesis of this disease seems to be insufficient. Environmental factors, e.g., viral and bacterial infections, food components, drugs, and toxins, which merely seem to trigger the onset of the disease, play an important role in its development [1,2].

According to the ISPAD (International Society for Paediatric and Adolescent Diabetes) Clinical Practice Consensus Guidelines 2018, T1DM is characterized by four stages:

Stage 1: Multiple islet antibodies, normal blood glucose, pre-symptomatic;
Stage 2: Multiple islet antibodies, raised blood glucose, pre-symptomatic;
Stage 3: Islet autoimmunity, raised blood glucose, symptomatic;
Stage 4: Long-standing type 1 diabetes [3].

Direct disease development is caused by an imbalance between CD4+ effector cells (Teffs) and Tregs. Teffs can promote beta-cell death and the production of immunoglobulins, which are markers of the autoimmune process. Regulatory T cells can be divided into thymus-derived naturally occurring Tregs (nTregs), Tregs induced in vivo (pTregs), and Tregs induced ex vivo with interleukin-2 (IL-2), plus transforming growth factor β (TGF-β) with or without retinoic acid or rapamycin (iTregs).

As specified by the Copenhagen model, the progressive autoimmune response is regarded to be responsible for the development of the disease. Numerous studies confirm that the absence of CD25+ cells in mice leads to the development of T1DM; the reconstitution of these cells results in the resolution of the symptoms of the disease [4,5,6,7,8].

Although there are many reports of in vitro and in vivo investigations, the role of natural Treg (CD4+CD25+) cells in the progress of T1DM is still unclear [4,5,6,7]. Natural Tregs play an important role in maintaining self-tolerance due to their influence on modifying the functional properties of other cells, e.g., CD4+ and CD8+ T lymphocytes, B lymphocytes, natural killers (NK) cells and dendritic cells (DCs). Among other things, they reduce interleukin 2 expression, destroy effector cells with granzyme B and perforin, and transform antigen-presenting cells (APCs) into suppressive cells. Natural Tregs are also able to produce cytokines that suppress the immune response, e.g., interleukin-10 (IL-10), interleukin-35 (IL-35) and TGF-β [9].

The dysfunction of the Tregs means that autoreactive T lymphocytes are not eliminated in the thymus, and the process of autotolerance does not develop. Instead, the effector cells produce cytokines such as interferon gamma (IFN-γ), interleukin-17 (IL-17) or tumor necrosis factor α (TNF-α) as well as granzyme or perforin, which damage the β cells of the pancreatic islets [10].

Several research teams have experimentally confirmed that T1DM develops due to a reduced number or impaired function of Tregs [7,11,12,13,14,15,16,17,18]. A reduction in the percentage of Tregs and impairment of their function was confirmed in a study on children with newly diagnosed diabetes. CD4+CD25+highCD127dim/−lymphocytes were regarded as Tregs [13]. A lower percentage of CD4+CD25+high cells were noted in T1DM-affected children. Furthermore, lower mRNA levels were observed for the genes of molecules and receptors that are characteristic for Treg lymphocytes, e.g., cytotoxic T-lymphocyte-associated protein 4 (CTLA-4), interleukin-10 receptor alpha (IL-10Rα), TGF-β1 and TGF-β2. This was also observed in transcription factors—signal transducer and activator of transcription 1 (STAT-1) and signal transducer 3 for receptor of the transforming growth factor beta (SMAD-3)—which may indicate the impairment of the function of this cell population in T1DM [15].

In addition to the number of Tregs, the percentage of apoptotic CD4+CD25+ cells were determined in children with T1DM that were diagnosed maximally in the last 6 months. The percentage of Tregs increased in children during T1DM remission. The exogenous insulin requirement and the concentration of peptide C in the serum correlated with an increased rate of CD4+CD25+ T-cell apoptosis [12,15]. Different results were obtained in patients with longer disease durations. The results of the investigation suggest that the remission phase is the only period suitable for intervention in the immune system [13].

The autoimmune reaction is active in prediabetes and decreases within a few years, after the clinical manifestation of T1DM. In this period, autoantigens, which are beta-cells of the pancreas, are destroyed, and the production of Tregs decreases [2,13].

## 2. Formation and Selection of Tregs

**Figure 1:**
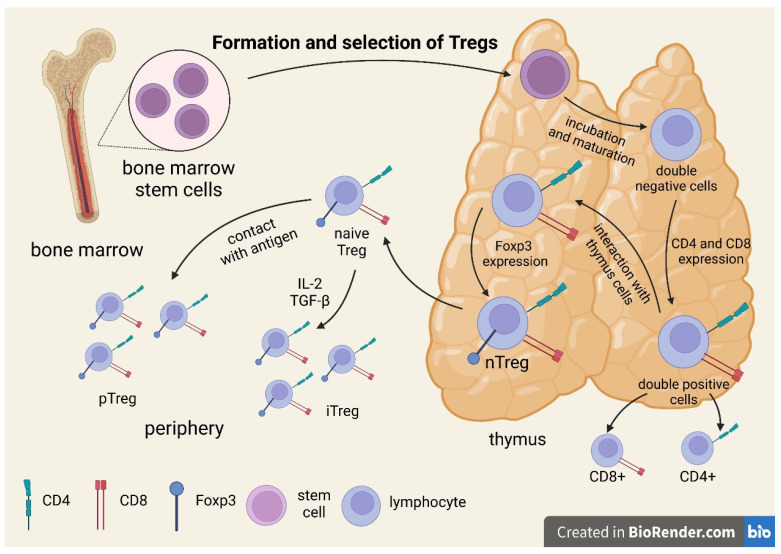
Formation and selection of Tregs.

All T cells originate from bone marrow stem cells and incubate and mature as a cell line in the thymus. At the double-negative (DN) stage, all T cells start as CD4-CD8-T-cell receptor (TCR) cells. At this stage, some cells change their T-cell receptor genes to form a unique functional molecule. After receiving the signal, they proliferate and simultaneously express CD4 and CD8, thus becoming double-positive cells. In this stage, they are selected by their interaction with cells in the thymus, starting with forkhead box P3 (Foxp3) transcription and becoming Treg cells, the well-described population of Foxp3+ Tregs. TCR expression is characteristic of Tregs. There are more diverse TCRs than effector T cells acting toward self-peptides [13,14,15,16]. The process of the formation and selection of Tregs is illustrated in Figure 1.

The Treg selection process depends on the affinity for interaction with the major histocompatibility complex (MHC). A T cell that receives a strong signal undergoes apoptotic death. When the signal is weak, the cell survives and can become a Teff. Only a T cell that receives an intermediate signal can develop into a Treg [19]. Tregs require CD28 co-stimulation and cell-surface protein ligand expression; the ligands bind to the CD28 family of receptors (B7.2) [17]. TGF-β is not necessary for Treg action in the thymus, as thymic Tregs (tTregs) from TGF-β-insensitive TGFβRII-DN mice have been found to be functional [17].

The development of tTregs is dependent on the processes involving TCRs during thymic development. Autoantigens are presented to the thymocytes in the medulla of the thymus. This presentation is dependent upon thymic mTEC (medullary epithelial cells) that randomly rearrange TRAs (tissue-restricted antigens) that are specific to various peripheral tissues. PGE (promiscuous gene expression) is a thymus-specific process that enables the selection of T-lymphocytes. As shown above, the intensity of the TCR signal determines the fate of thymocytes [19].

The cortex of the thymus gland is the site of positive selection for conventional T cells and early Tregs. Cortical thymic epithelial cells (cTECs) produce their own peptides through a characteristic set of proteases [19].

The role of TCR specificity in determining thymocyte conversion to Treg and conventional T lymphocyte (Tconv) cells remains unclear. It seems that, in TCR interactions, a preliminary decision is made as to whether a given thymocyte will be capable of becoming a Treg cell. Conversely, Treg cells may recognize a wide range of foreign antigens and may show overlap with Tconv cells, and TCR-dependent processes may be complementary to processes in which TCRs are not involved and that affect Treg-cell precursors [20,21].

Studies have shown that regulatory cells retain the ability to proliferate in the presence of IL-2, interferon beta (IFN-β), interleukin-15 (IL-15) and secreted proinflammatory cytokines, although the ability is substantially lower than that of induced Treg (CD4+CD25-) lymphocytes [15,16,17]. The decrease in Treg functionality may be due to the deficiencies of TCR, the IL-2/ IL-2Rα pathway and suppressor mechanisms such as CTLA-4 [22].

For the maintenance of development and functionality, the transcriptional factor Foxp3 seems to be pivotal. It has been reported that mutations in Foxp3 in mice and its orthologue in humans lead to a phenotype with severe autoimmune disease; these are known as the scurfy mutation in mice and polyendocrinopathy, enteropathy, immune dysregulation, and X-linked syndrome (IPEX) in humans [16,23,24]. In mice with a deficiency in the IL-2 and IL-2R subunits, IL-2 is a crucial cytokine necessary for the induction of Foxp3 expression, differentiation of FoxP3+ Tregs in the thymus, and their maintenance with their suppressor ability in tissues [25,26]. IL-2 deprivation may even lead to loss of Foxp3 expression and the conversion of Tregs into pathogenic Teff cells [17,23,24,25,27,28]. It has been determined that the activity of Foxp3 is mainly influenced by genetic changes. Multiple single-nucleotide polymorphisms (SNPs) located in coding or non-coding regions of the gene FOXP3 directly regulate, while SNPs such as those in IL2RA (CD25) and protein tyrosine phosphatase non-receptor type 2 and 22 (PTPN2 and PTPN22) indirectly regulate Foxp3 via signal transducer and activator of transcription 5 (STAT5). Moreover, the expression of Foxp3 is regulated by metabolism and the glycolytic process. MicroRNA (miRNA) regulates the mRNA for Foxp3 by binding it and inducing its cleavage, destabilization and targeting to stress granules, while the Foxp3 protein itself is regulated through post-translational modifications such as phosphorylation, acetylation and ubiquitination. The above factors, occurring at various stages, lead to the dysregulation of Tregs and loss of the self-control process, resulting in an autoimmune disease [29].

Foxp3+ Tregs especially exert their suppressive effects in a cell-contact-dependent manner. Contact with APCs, e.g., DCs, through surface-expressed inhibitory molecules, such as programmed death-1 ligand (PD-L1) or CTLA-4, can either exclude Teffs from contact with DCs or possibly change the DC phenotype to a tolerogenic nature. CTLA-4 or PD-L1 is spontaneously expressed constitutively in Foxp3+ Tregs. CTLA-4 or PD-L1 is expressed only after activation in Teffs. CTLA-4 is considered to outcompete CD28 in binding the costimulatory molecules CD80 and CD86 in APCs, thus decreasing their capacity to activate Teffs [26,30,31]. The involvement of CTLA-4 can also induce the production of the immunosuppressive molecule indoleamine 2,3-dioxygenase (IDO) by DCs. IDO induces the production of kynurenine, a pro-apoptotic metabolite, from tryptophan catabolism, suppressing Teffs, but it also functionally changes DCs to secrete immunoregulatory cytokines (e.g., IL-10 or TGF-β), which change adjacent T cells to cells with a regulatory phenotype [32]. Suppression can be mediated by FoxP3+ Tregs through the secretion of immunomodulatory mediators such as IL-35, by metabolic disruption via adenosine generated by the ectoenzymes CD39 and CD73, and by cytolysis in a granzyme B/perforin-dependent manner [33,34,35,36,37]. A comparison of the expression of Treg microRNAs selected from the peripheral blood in T1D patients undergoing IT shows the impairment of the in vitro suppressive functions of Tregs isolated from the pancreas or pancreatic lymph nodes (PLN). It was observed that miR-125a-2p was overexpressed in Tregs isolated from PLN. This situation was inversely correlated with the expression of the type 2 CC chemokine receptor, which is essential for Treg migration to sites of inflammation, suggesting that PLN may be the primary site of Treg dysfunction in T1D [24].

Other types of Tregs, their features, and their contribution to the development of type 1 diabetes are presented in Table 1.

## 3. Mechanism of the Suppressive Action of Treg Lymphocytes

**Figure 2:**
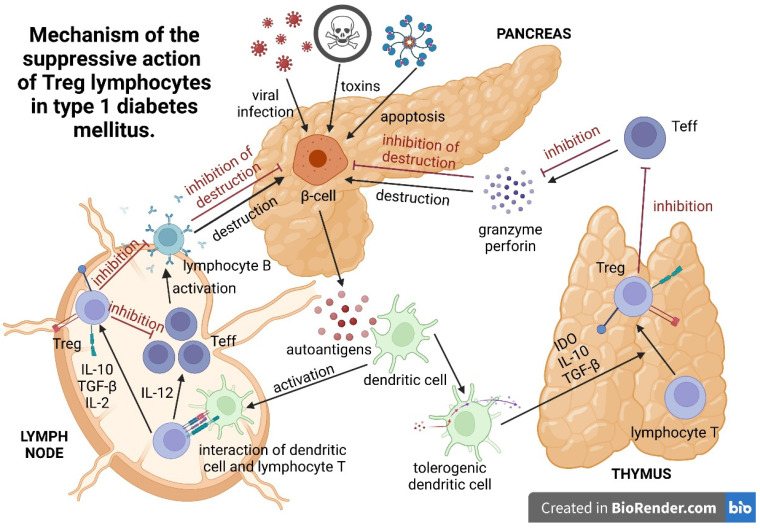
Mechanism of the suppressive action of Treg lymphocytes in type 1 diabetes mellitus.

Peripherally induced Tregs exist in organisms as peripheral and local Tregs, accumulating in inflamed tissue (Figure 2). Pancreas-residing Tregs play a main role in the suppression of pancreatic islet inflammation. In their study, Jonuleit et al. proved the need for direct Treg-lymphocyte–effector-lymphocyte contact to achieve a suppressive effect. The reduction of proliferation and lower activation of conventional T-helper cells depends on direct cell–cell contact. Cytokines, such as IL-10 and TGF-β, produced by lymphocytes play a minor role in this process [49]. It is likely that the cell–cell contact between Treg lymphocytes and CD4+ and CD8+ lymphocytes, monocytes, and antigen-presenting B cells is related to the death receptor (FAS)-independent synthesis of perforins, CD18, and granzyme A [50].

Additionally, it has been shown that, upon direct contact with the CD4+CD25+ cell, the effector lymphocyte enters the anergy state and acquires the ability to inhibit other CD4+CD25- cells. In in vitro cell cultures, considerable amounts of IL-10 and TGF-β released by CD4+CD25- lymphocytes were observed in this reaction. The two-step inhibition of effector cells by CD4+CD25+ lymphocytes has been called infectious tolerance [49].

The expression of the CTLA-4 molecule increases on the surface of an activated CD4+CD25+ lymphocyte and persists for several weeks [49]. The glucocorticoid-induced TNF (GITR) receptor, also referred to as TNFRSF18, is another known surface receptor, influencing the suppressor function of CD4+CD25+ cells. It is primarily present on CD4+CD25+ and CD4+CD25+CD8- cells. Investigations conducted in a murine model have shown reductions in the suppressive properties of Tregs and development of autoimmune diseases after stimulation with anti-GITR monoclonal antibodies [17]. The adoptive transfer of Tregs decreased the intercellular adhesion molecule 1 (ICAM-1) in the diabetic pancreas and decreased the production of IFN-γ [51].

In their study, Li Zhou et al. indicated that iTregs could inhibit Th1 cells via FasL-dependent cytotoxicity. This may be mediated by decreased Tc1-cell proliferation, increased Tc1-cell apoptosis, inhibited Tc1-cell differentiation or reduced Tc1-cell infiltration [47].

## 4. Treg Cells in the Treatment of T1DM

The key to the causal treatment of T1DM lies in the prevention of the early loss of pancreatic islets in predisposed individuals, the regeneration of the pancreatic islets during the remission phase, or pancreatic islet transplantation in the chronic form of the disease. These three targets are potentially achievable through regulatory lymphocyte therapy [52].

However, the implementation of Treg-cell treatment requires further research. The first problem posed to researchers is the precise isolation of patients’ autologous regulatory cells. CD4, CD25, and CD127 seem to be the most specific markers [53]. Over 96% of the CD4+CD25+CD127-/low cell population expresses the intracellular transcription factor Foxp3 [54]. Serr et al. noted problems in identifying Tregs in the peripheral blood in humans, despite the cells being characterized as CD25+ CD127 low FOXP3+, due to the different degrees of activation, functionality, and lack of homogeneity in composition, e.g., the discrepant expression of CD45RA (the presence of which was associated with stronger suppressive abilities) [28].

Serr et al. also demonstrated a reduced incidence of insulin-specific Tregs in the blood of children at risk of diabetes, especially at the onset of autoimmunity against pancreatic islets, while a higher percentage of these lymphocytes were associated with slower progression to clinically overt type 1 diabetes [28].

Another problem is ensuring the Tregs’ effectiveness in the suppression of a specific population of activated lymphocytes. The key to achieving this objective is the application of antigen-specific Treg cells to pancreatic islets. However, the population of regulatory cells circulating in human peripheral blood accounts for less than 5%, with pancreatic islet antigen-specific Treg cells occurring at a frequency of 1:150,000 cells [55]. The final but probably most important question is the effectiveness and safety of the innovative therapy. An opportunity for the effective suppression of the autoimmune reaction and reconstruction of damaged beta cells seems to be offered by the application of a combination therapy based on the infusion of Tregs and administration of an anti-lymphocyte serum or anti-CD3 antibodies, which have been shown to exhibit a certain degree of effectiveness in slowing the progression of the disease [56].

### 4.1. Treg-Cell Transplantation

Attempts have been made to use Treg cells in the prevention and treatment of T1DM in animal models. Experimental animals were treated with naïve Treg cells, specifically recognizing pancreatic beta cells, followed by virgin and activated T cells specific for pancreas beta-cell antigens. Promising results were obtained, which proved that T cells were effective in the treatment of a severe form of T1DM in an animal model [57,58].

Brusko and Bluestone successfully used polyclonal Tregs in the therapy of patients with T1DM [59,60].

Another form of therapy is to use Tregs transduced with autoantigen-specific T-cell receptors in T1D. Researchers used a protocol for lentiviral TCR gene transfer, which recognizes autoantigens associated with type 1 diabetes (GAD_555–567_), to induce antigen-specific tissue tolerance to stop β-cell destruction. They showed that de novo Treg avatars suppress antigen-specific and bystander responder T-cell (Tresp) proliferation in vitro in a process that requires Treg activation, which suggests an important role for antigen-specific Tregs for use in attenuating β-cell autoimmunity as a new therapeutic option for type 1 diabetes [61]. Studies in non-obese diabetic (NOD) mice have shown that a small number of antigen-specific Treg cells (e.g., 2000) were required to prevent (and sometimes reverse) changes in type 1 diabetes [62,63]. The difficulty is in adequately identifying high-affinity and autoantigen-specific TCRs capable of transducing Tregs [64].

Marek-Trzonkowska et al. conducted investigations on the use of Treg cells in T1DM children. The study involved a group of 12 children treated experimentally with a single or double infusion of autologous Tregs at a total dose of 30 × 10^6^/kg. No serious side effects of the treatment were reported. Within a year, the C-peptide level increased, and the basic exogenous insulin requirement declined in the treated patients [65,66,67]. Lengthy observation of these patients indicated that the infusion of autologous Treg cells should be repeated for the long-term remission of T1DM [66,67]. Notably, proinsulin-specific T-regulatory cells were found to control the immune responses in the disease [67].

Gliwinski et al. analysed the subclasses of T lymphocytes in newly diagnosed and long-term diabetic patients. In the latter group, they singled out patients previously treated with polyclonal Tregs to investigate the effect of this therapy on lymphocyte subsets. The proinsulin peptides and glutamic acid decarboxylase served as autoantigens. Immunosenescence-like changes of T lymphocytes caused by the expansion of these cell clones and that exacerbate the course of type 1 diabetes have also been found. It was also shown that proinsulin-specific T-regulatory lymphocytes from polyclonal preparations counteracted the described changes by increasing polyclonality or lengthening lymphocyte telomeres, and thus decreased the immune response in diabetes [68].

Sodré et al. proposed a therapy with BB-Cl-amidine treatment, which, by inhibiting a peptidylarginine deminase (PAD), reduced the protein citrunalization involved, and it prevented diabetes development in NOD mice. In their study, they showed that BB-Cl-amidine resulted in an induced shift from Th1 to Th2 cytokines in the serum and an increase in the frequency of Tregs in the blood and spleen, as well as a decrease in the level of autoantibody formation against citrullinated 78-kDa glucose-regulated protein (GRP78) and reduction of idiopathic NETosis of bone marrow neutrophils. In the pancreas, insulin production was preserved, and, at the same time, inflammatory infiltration was reduced by decreasing the effector memory CD4+ T cells and IFN-γ-producing CD4+ and CD8+ T cells [69].

The therapeutic application of regulatory T cells in T1DM is presented in Table 2.

### 4.2. Induction of Tregs

In addition to the infusion of regulatory lymphocytes in the immunotherapy of T1DM, researchers have attempted to increase the Treg population through cytokine interactions, pharmacotherapy, and stimulation with other cell subpopulations. The IL-2 cytokine has high affinity for Treg cells due to the high expression of CD25+, which is necessary for proliferation. It has been demonstrated in an animal model that the presence of IL-2 increases the percentage of regulatory lymphocytes, inhibits inflammatory-cytokine release, and prevents the development of T1DM [68,70,71]. In a few clinical studies involving treatment with IL-2 in T1DM-affected patients, the percentage of Treg lymphocytes increased, and the therapy proved to be safe [70,72].

The induction of self-tolerance with Tregs can be achieved with the use of dendritic cells with an anti-inflammatory phenotype. There are three strategies for realizing interactions of dendritic cells with Treg cells: (1) the infusion of tolerogenic dendritic cells, (2) the in vitro proliferation of Treg lymphocytes via interactions with dendritic cells, and (3) the in vivo proliferation of Treg lymphocytes via interactions with dendritic cells [73]. Tolerogenic dendritic cells can be induced by cytokines: granulocyte/macrophage colony stimulating factor (GM-CSF), granulocyte colony stimulating factor (G-CSF), IL-10, and TGF-β; drugs: dexamethasone, vitamin D3, and rapamycin; or exposure to the CTLA-4 membrane receptor and oligonucleotides [73]. In T1DM patients, the application of anti-thymocyte globulin combined with G-CSF increased the percentage of regulatory T cells, thus protecting the function of beta cells [73,74,75,76].

L. Zhou et al. investigated the preventive and therapeutic roles of induced Tregs in T1DM. They used Tregs induced ex vivo by IL-2 and TGF-β on a T1DM model induced by the repeated injection of low doses of streptozotocin (STZ), which induces pancreatic beta-cell damage through an autoimmune reaction involving lymphocytes that faithfully reflects the pathogenetic mechanism in T1DM. The authors showed that iTregs suppressed Tc1 cells—CD8+ cells that secrete IFN-γ and TNF-α—and mainly contributed to the cytotoxic activity of CD8+ T cells in T1DM through TGF-β-mediated combinational action on mammalian target of rapamycin (mTOR) and T-cell factor 1 (TCF1) signalling, while rebalancing endogenous IFN-γ-producing CD8 T cells. iTregs ameliorate the progression of T1DM and can delay the occurrence of T1DM by lowering blood glucose levels, reducing islet damage and improving β-cell function and morphology [47].

The suppressor effect of nTregs or iTregs producing large amounts of IL-10 can also be achieved by “vaccination” with a small amount of immunogenic peptides specific for the autoimmune disease [74,75]. In the case of diabetes, this effect can be achieved using insulin/proinsulin B9/23 epitopes (proteins 9–23 of the insulin beta chain) or C19-A3 (CA proinsulin chain junction) [74,75,77,78,79]. Therapy based on the application of proinsulin peptides (C19-A3) in patients with newly diagnosed T1DM (<100 days) appeared to be safe and well tolerated. It was also effective, as there was no increase in the insulin requirement and no decrease in blood C peptide for 12 months [78]. Although the results of these studies are encouraging, this strategy carries a risk of the induction of effector CD4+ and cytotoxic CD8+ cell responses [78,79,80,81].

The results of F. Russo et al. also indicate a promising therapeutic option. They demonstrated that the administration of a lentiviral vector enabling the expression of insulin B chain 9–23 (InsB9-23) in hepatocytes arrested B-cell destruction in prediabetic NOD mice by generating InsB9-23—specifically in FoxP31 Tregs—and it could also improve the efficacy of allogeneic islet transplantation [82].

Oral insulin administration has been attempted in patients as a form of primary and secondary prevention of type 1 diabetes mellitus based on the assumption that insulin, as an antigen, will induce immunological tolerance. Assfalg et al. conducted a clinical trial involving children who were 6 months to 3 years of age with a first-degree relative with type 1 diabetes and with the HLA DR4-DQ8 genotype. During the 12-month follow-up, the children received daily insulin or placebo, and the response of regulatory T cells was checked. The expected immune response was not obtained, but it was shown that, in children with a susceptible INS genome, there was the possibility of modifying the immune system’s response to oral insulin. An increased ability of CD169+ monocytes to activate naïve insulin-reactive T cells was also demonstrated [83].

J. Sun et al. evaluated the efficacy of kynurenine (Kyn), an endogenous substance that can inhibit NK and T cells’ proliferation and promote the differentiation of naïve T cells into Tregs, as a novel suppressive adjuvant. Co-immunization with a Kyn and GAD65 phage vaccine resulted in decreased GAD65-specific T-cell proliferation, suppressed DC maturation, increased CD4+ CD25+ Foxp3+ Treg cells, regulated the Th1/Th2 imbalance, and increased the secretion of Th2 cytokines and prevented hyperglycemia in 60% of the mice of a NOD mouse model for at least one month [84].

The oral administration of all-trans-retinoic acid and transforming growth factor-β- for the amelioration of T1DM was investigated in animals. The result of this research was the achievement of prolonged stimulation of regulatory immune cells. Both tolerogenic dendritic cells (tolDC) (CD11c^+^CD11b^−^CD103^+^) and Tregs (CD4^+^CD25^high^FoxP3^+^) were enhanced. The Th1 response was inhibited, and a reduced rate of immune-cell infiltration into the pancreatic islets of mice was observed [85,86].

The defect in the intestinal microbiota, both in the animal model and in patients with diabetes, is irreversibly associated with the development of autoimmunity, although changes in the intestinal microbiota were related to the progression of the disease rather than to the initiation of autoimmunity [87]. The oral administration of lyophilized probiotic bacteria (Bifidobacteria, Lactobacilli, and Streptococcus salivarius strains) to NOD mouse strains induced the generation of IL-10-producing iTreg-Tr1 lymphocytes by gut-associated lymphoid tissue (GALT). This prevented the destruction of beta cells and delayed the development of the signs of clinical diabetes. Suppressive T cells induced in the gastrointestinal tract retained the ability to migrate to the periphery and to target organs by suppressing the diabetogenic effector lymphocytes [87].

The role of innate type 3 lymphoid cells (ILC3) in the pathogenesis of T1DM has recently been underlined. ILC3, residing in the GALT, secretes IL-17 and GM-CSF, which activate immune cells to combat potentially pathogenic microorganisms, and which also produce IL-22. Moreover, ILC3 secretes IL-2 and promotes Treg generation and function. Therefore, new therapeutic options for T1DM involving stimulating the free fatty acid receptor (FFAR2) using available selective synthetic agonists are being sought. It is suggested that, upon enhancing ILC3 function in this manner (for example, the application of aryl hydrocarbon receptor (AhR) ligands, vitamin A, vitamin D, or short-chain fatty acids (SCFAs)), the immune response against pancreatic B cells will be weakened [88].

Iranian researchers used a local methanolic extract of Sambucus ebulus (SE), an anti-inflammatory agent used in Iranian traditional medicine, on the STZ-induced-T1DM mouse model. In mice that received SE at a dose of 400 mg/kg, the symptoms of diabetes were less pronounced, the insulin content in the islets increased as did the level of IL-10, and the levels of IFN-γ and IL-17 decreased. The main protective contribution of SE relates to the increases in the levels of CD4+ and CD8+ T cells and the increase in the number of Treg cells, which resulted in a reduction in inflammation in the pancreatic islets [89].

Interesting investigations were conducted by Duan et al., who observed that the treatment of NOD mice with metformin significantly mitigated autoimmune insulitis and substantially decreased the number of proinflammatory IFN-γ+ as well as IL17+ CD4 T cells in the spleen. However, a significantly increased percentage of regulatory IL-10+ and Foxp3+ CD4 T cells was observed [90]. Potential therapeutic functions of antibodies to IL2 were suggested by Spangler et al. [91].

### 4.3. Pancreatic Islet Transplantation

Whole-pancreas transplantation as a method of T1DM treatment has been associated with technical difficulties and numerous complications. Conversely, islet transplantation is an easier procedure to perform, but the autoimmune rejection of the allograft is a problem.

J.-R. Lin et al. conducted studies using valproic acid (VPA), which is usually used as an anti-epileptic drug, and they established that VPA could be successful in protecting islet grafts and prolonging graft survival in islet transplantation. The study was performed in a NOD mouse model using VPA at a dose of 400 mg/kg s.c. VPA treatment prolonged the survival of islet grafts after islet transplantation and increased the IL-4-producing CD4 T-cell and Treg-cell populations in NOD recipients, and VPA induced Treg differentiation from naïve CD4 T cells by increasing the expression of the transcription factor STAT5 and histone 3 (H3) acetylation. However, VPA has many side effects such as hepatotoxicity, hyperammonaemia, weight gain and insulin resistance. To avoid this, the authors observed the same effectiveness with the use of an adoptive transfer of in vitro VPA-induced regulatory T cells, which also prolonged islet-graft survival [92].

The recent study conducted by Herold et al. proposed the prophylactic use of teplizumab (anti-CD3 antibody) in relatives at risk for type 1 diabetes [93].

Despite many uncertainties, regulatory lymphocytes have recently become a potential opportunity for the safe and effective causal therapy of T1DM.

One weakness of Treg infusion or activation for the therapy of T1DM is the necessity of the stable continuation or replication of the therapy. Long-term observations indicate that the effects of these methods are transient [67]. The authors suggested that the effect of Treg activation or transplantation may be better in the earlier phase of T1DM, especially in the preclinical phase. The development of early diagnosis methods may allow the identification of patients in the early preclinical phases of T1DM. Immune intervention before damage to a large number of beta cells may be a method for the prevention of T1DM development.

## 5. Conclusions

Treg cells play important roles in the limitation of the development of T1DM.

The activation or application of Tregs may be more effective in the early stages of T1DM development, especially in pre-symptomatic T1DM.

The therapeutic use of Treg cells in T1DM is promising but needs long-term observations in a large group of patients.

## Figures and Tables

**Table 1:** Characteristics of Treg cells [17,36,37,38,39,40,41,42,43,44,45,46,47,48].

Regulatory T Cells	Foxp3+Tregs	Tregs of B Cells (B-Cell-Induced Tregs)	Th3	Tr1	CD8^+^ Tregs	nTregs	iTregs
**Selection place**	Naïve Tregs—thymus. Peripheral Tregs—peripheral tissues and MALT (mucosa-associated lymphoid tissue). Induced Tregs—in vitro-induced Tregs	Spleen MALT	Gut MALT	Spleen Lymph node	Spleen Lymph node	Thymus	In vitro/ Peripheral tissues
**Phenotype**	CD4^+^, CD25^+^, Foxp3^+^, CTLA-4^+^	CD4+CD25+Foxp3- LAG 3+ ICOS+PD1+GITR+ OX40+	CD4+CD25-Foxp3- LAP+	CD4^+^CD25^−^ Foxp3^+^, CD49b^+^, LAG3^+^, CD226^+^	CD25^+^, Foxp3^+^ CD28-	CD4^+,^ CD25^+^, Foxp3^+^	CD25^+^, Foxp3^+^
**Mechanism of suppressive action**	Direct cell–cell contact Granzyme-B-dependent formation of TGF beta	IL-10	TGF beta IL-10 (strong)	IL-10 (strong) TGF beta CTLA4 CD226	IL-4, IL-5, IL-10	TGF beta IL-2 CD28	TGF beta
**Role in T1DM development**	Reduction in the functional capacity of Foxp3+Treg populations contributes to disease development in type 1 diabetes	Immunomodulatory effect?	Th3 cells probably originate from naïve T cells as a result of stimulation with immature dendritic cells (iDCs), presenting various antigens, including autoantigens. Such stimulation results in the in vivo and in vitro formation of anergic cells with suppressive properties.	It has been shown that newly diagnosed T1DM patients and their first-degree relatives have fewer IL-10-secreting cells than healthy controls; this deficit in Treg function is amplified by an increased Teff function, which is reflected by increased antigen-specific IFN-c secretion.	The main triggers of β-cell autoimmunity	Inhibiting the immune response of effector T cells and maintaining immune tolerance	Inhibition of Th1 cells by a FasL-mediated cytotoxic effect Decreased Tc1-cell proliferation, increased Tc1-cell apoptosis, decreased Tc1-cell infiltration and/or inhibition of Tc1-cell differentiation

**Table 2:** Therapeutic application or induction of Tregs in T1DM.

Methods of Therapy	Authors	Number of Patients	Results
**Transplantation of autologous Tregs**	Bluestone 2015 (42) Marek-Trzonkowska et al., 2012, 2014, 2016, 2020 (43–45) Brusko 2008 (58)	14 12	The therapy resulted in beta-cell regeneration, insulin production, and a strong decrease in therapeutic insulin intake. T1DM development was associated with changing proportions of naïve and memory Tregs and slowly increasing proinflammatory activity, which was only partially controlled by the administered Tregs. The authors suggest that the therapy should be administered early to protect the highest possible mass of islets and to utilize the preserved content of Tregs in the earlier phases of T1DM. The therapy extended remission of the T1MD (honeymoon) and decreased the doses of insulin necessary for treatment.
**Low dose of IL-2**	Hartemann et al., 2013 (47) Todd et al., 2016 (48) Rosenzwaig 2015, 2020 (49, 50)	24 40 24	The authors defined a well-tolerated and immunologically effective dose range of IL2 for application to type 1 diabetes therapy and prevention. Early intervention with IL2 could help to re-establish a proper immune milieu and slow down or even reverse the pathological processes in T1DM. This therapy may improve maintenance of induced C-peptide production at 1 year. The adverse effects were influenza-like syndrome and injection-site reactions.
Induction of Tregs by tolerogenic DCs			
**Anti-thymocyte globulin and G-CSF (ATG/GCSF)**	Haller et al., 2015, 2017, 2019 (52,53,64)	17	Immune responses returned to normal upon withdrawal of therapy.
**CTLA-4-Ig** **(abatacept)**	Orban et al., 2013 (65)	112	Co-stimulation modulation with abatacept slowed the decline of beta-cell function and improved HbA1c in recent-onset T1DM. The beneficial effect was sustained for at least one year after cessation of abatacept infusions or three years from T1DM diagnosis.
**Methyldopa**	Ostrov et al., 2018 (66)	20	Methyldopa specifically blocked DQ8 in patients with recent-onset T1D, highlighting the relevance of blocking disease-specific MHC class II antigen presentation to treat autoimmunity.
**Alum formulated glutamate decarboxylase**	Elding Larsson 2018 (67)	50	The subcutaneous prime and boost administration of GAD-Alum was safe but did not affect progression of T1DM.
**Vitamin D3**	Piekarski 2012 (68)	90	Alfadiol, an analogue of vitamin D3, increased or maintained the value of C-peptide during the annual monitoring as compared with baseline values. This therapy extended the honeymoon in children with newly diagnosed T1DM.
**Autologous tolerogenic dendritic cells**	Giannoukakis 2011 (54)	10	The autologous tolerogenic dendritic cells in patients between 1 and 5 years of T1DM upregulated the frequency of B220+CD11c-B-cells, but the average insulin dose in patients remained unchanged.
Induction of Treg by tolerogenic peptides			
**Oral insulin B**	Orban et al., 2010 (56) Krischer 2017 (69)	12 560	Oral insulin (7.5 mg/day) did not delay or prevent the development of type 1 diabetes. A higher dose (67.5 mg/day) was reported to produce protective insulin-responsive regulatory T-cell responses in genetically at-risk young relatives.
**Proinsulin C19-A3**	Alhadj et al., 2017 (5)	27	Proinsulin peptide therapy restored immune tolerance in preclinical phase of T1DM but did not accelerate the decrease in beta-cell function.
**Induction of Tregs by p53 activation**	Pellegrino (70)	10	Due to the Teff dysregulation upon p53 activation, molecules promoting p53 cannot be part of the therapy for T1DM.
**HSCT (human stem-cell transplantation)**	Li 2012 (71) Zhang 2012 (72) Gu 2012 (73) D’Addio 2014 (74) Carlsson 2014 (75)	13 9 28 65 20 1	Human stem-cell transplantation is a safe and promising method for T1DM treatment and leads to an increase in C-peptide and insulin secretion. This method is more effective in the early stages of T1DM. Patients diagnosed in ketoacidosis at diagnosis have minimal chance for beta-cell recovery.

## Data Availability

Not applicable.
